# Centriole polarisation to the immunological synapse directs secretion from cytolytic cells of both the innate and adaptive immune systems

**DOI:** 10.1186/1741-7007-9-45

**Published:** 2011-06-28

**Authors:** Jane C Stinchcombe, Mariolina Salio, Vincenzo Cerundolo, Daniela Pende, Maurizo Arico, Gillian M Griffiths

**Affiliations:** 1 Cambridge Institute for Medical Research, University of Cambridge, Hills Road, Cambridge CB2 0XY, UK; 2 Human Immunology Unit, Weatherall Institute for Molecular Medicine, University of Oxford, John Radcliffe Hospital, Oxford, OX3 9DS, UK; 3 National Institute for Cancer Research, Largo Rosanna Benzi 10, I-16132 Genoa, Italy; 4 Department of Oncology and Pediatrics, Azienda Hospital, Meyer University, I-50139 Florence, Italy

## Abstract

**Background:**

Cytolytic cells of the immune system destroy pathogen-infected cells by polarised exocytosis of secretory lysosomes containing the pore-forming protein perforin. Precise delivery of this lethal hit is essential to ensuring that only the target cell is destroyed. In cytotoxic T lymphocytes (CTLs), this is accomplished by an unusual movement of the centrosome to contact the plasma membrane at the centre of the immunological synapse formed between killer and target cells. Secretory lysosomes are directed towards the centrosome along microtubules and delivered precisely to the point of target cell recognition within the immunological synapse, identified by the centrosome. We asked whether this mechanism of directing secretory lysosome release is unique to CTL or whether natural killer (NK) and invariant NKT (iNKT) cytolytic cells of the innate immune system use a similar mechanism to focus perforin-bearing lysosome release.

**Results:**

NK cells were conjugated with B-cell targets lacking major histocompatibility complex class I 721.221 cells, and iNKT cells were conjugated with glycolipid-pulsed CD1-bearing targets, then prepared for thin-section electron microscopy. High-resolution electron micrographs of the immunological synapse formed between NK and iNKT cytolytic cells with their targets revealed that in both NK and iNKT cells, the centrioles could be found associated (or 'docked') with the plasma membrane within the immunological synapse. Secretory clefts were visible within the synapses formed by both NK and iNKT cells, and secretory lysosomes were polarised along microtubules leading towards the docked centrosome. The Golgi apparatus and recycling endosomes were also polarised towards the centrosome at the plasma membrane within the synapse.

**Conclusions:**

These results reveal that, like CTLs of the adaptive immune system, the centrosomes of NK and iNKT cells (cytolytic cells of the innate immune system) direct secretory lysosomes to the immunological synapse. Morphologically, the overall structure of the immunological synapses formed by NK and iNKT cells are very similar to those formed by CTLs, with both exocytic and endocytic organelles polarised towards the centrosome at the plasma membrane, which forms a focal point for exocytosis and endocytosis within the immunological synapse. We conclude that centrosomal polarisation provides a rapid, responsive and precise mechanism for secretory lysosome delivery to the immunological synapse in CTLs, NK cells and iNKT cells.

## Background

Cells of the immune system defend the body against pathogens. The immune response can be broadly divided into the innate and adaptive responses, with cells of the innate system providing an initial broad-specificity response to pathogens and cells of the adaptive system providing a later, more specific response. Differing recognition patterns of innate versus adaptive cells is accomplished via the use of distinct receptors. Cytotoxic T lymphocytes (CTLs), natural killer (NK) cells and invariant NKT (iNKT) cells are cytolytic cells of the immune system that are able to destroy cells infected with subsets of intracellular pathogens. NK and iNKT cell receptors provide broad recognition for the innate response via a limited repertoire of activating and inhibitory NK cell receptors (for NK cells) [1] or a semi-invariant NK T-cell receptor (TCR) (for iNKT cells) [2], while CTLs provide the highly specific recognition of the adaptive response by use of many different TCRs. Although NK cells, CTLs and iNKT cells all use different receptors to recognise their targets, they deliver a virtually identical lethal hit that destroys the target cell recognised [3]. All three cytolytic cell types contain secretory lysosomes containing the pore-forming protein perforin as well as a series of serine proteases termed 'granzymes'. Once released from the cytolytic cell, perforin forms transmembrane pores that enable the granzymes to enter the cytoplasm of the target cell and cleave substrates that trigger rapid apoptosis. Target cell death proceeds within minutes.

Precise delivery of the lethal hit is vital to ensuring that only the recognised target is destroyed while innocent bystander cells are left unharmed. CTLs use an unusual mechanism to direct secretion to the point where the T cell recognises its target [4]. CTLs identify target cells via TCRs, which recognise pathogen-derived peptides bound to major histocompatibility complex class I (MHC I) 721.221 cell molecules on the surface of the target cell and activate downstream signalling pathways. This triggers a number of events. First, receptors involved in adhesion and recognition segregate into a highly organised structure at the interface between lymphocyte and target, which was first identified in T-helper cells and has become known as the 'immunological synapse' [5-8]. T-cell receptors involved in recognition cluster to form a central supramolecular activation complex (cSMAC) surrounded by a ring of integrins involved in cell-cell adhesion to form a peripheral SMAC (pSMAC) [5]. Second, and unusually, the centrosome moves up to and 'docks' with the plasma membrane opposite the target. Docking always occurs at the centre of the immunological synapse next to the cSMAC [4] containing the activated TCRs. Since secretory lysosomes travel along microtubules, which emanate from the mother centriole, the movement and precise localisation of the centrosome in turn directs secretory lysosomes to a very defined point for exocytosis as they move down the microtubules to the polarised centrosome. Secretion occurs within the pSMAC and immediately adjacent to the cSMAC [9]. Centrosomal polarisation is exquisitely sensitive to TCR activation and responds to relatively weak signals, while a higher threshold of signalling is required to recruit secretory lysosomes to the docked centrosome [10].

Release of cytolytic proteins is confined to a secretory cleft that forms in an area of otherwise tight membrane contact and provides a confined space in which perforin and other lytic proteins can be kept concentrated for attack on the target [9]. The cleft forms regardless of lytic protein secretion, since clefts have been observed in CTLs from mice with genetic mutations that prevent exocytosis [11]. The mechanisms that trigger cleft formation are not understood. However, morphological studies on CTLs have shown that the centrosome contacts the plasma membrane to the side of, but not directly opposite, the secretory cleft [9,12]. In this way, microtubules emanating from the centrosome pass under the plasma membrane at the secretory cleft and thus direct the secretory lysosomes to the cleft membrane as they travel towards the polarised centrosome.

Previous studies of CTLs have revealed that not only does the centrosome direct polarisation of the secretory lysosomes to the immunological synapse, but the Golgi complex [4,13-15] and the recycling endosomes also polarise to the point of centrosomal contact with the plasma membrane [4,16]. In this way, centrosome positioning directs membrane traffic, and the synapse becomes a focal point for both exocytosis and endocytosis. The immunological synapse bears a number of striking similarities to primary cilia where the centrosome also migrates right up to the plasma membrane and forms the site of initiation for cilia formation [17-20]. Curiously, the Golgi complex [17] and recycling endosomes [21] also polarise to the site of cilia formation and to the flagella pocket [22], and the plasma membrane around the cilia also becomes specialised in exocytosis and endocytosis [12].

While the role of the centrosome in directing polarised secretion has been well documented in CTLs, little is known about whether the centrosome contacts the plasma membrane in other cytolytic cell types of the immune system with perforin-containing secretory lysosomes. Both NK and iNKT cells polarise their secretory lysosomes towards the immunological synapses formed with antigen-presenting cells and can kill target cells, and the strength of signalling can modulate the degree of granule polarisation and killing of agonist-pulsed targets for iNKT cells [23]. In this study, we asked whether the centrosome directs secretion towards a cleft in NK and iNKT cells, using high-resolution electron microscopy (EM).

## Results and discussion

### The centrioles of NK and iNKT cells polarise tightly to the plasma membrane in the immunological synapses formed with their targets

The docking of centrioles with the plasma membrane is most clearly visualised by imaging of contact sites using high-resolution EM. To investigate whether the same tight polarisation of centrosomes to the plasma membrane seen in CTLs occurs in other cytolytic cells of the immune system during target cell destruction, we therefore used high-resolution EM to examine the contact sites of NK cells conjugated to MHC I-deficient B cells and iNKT cells conjugated to lipid-pulsed, CD1d-bearing target cells [24].

Since the secretory lysosomes of CTLs and NK and iNKT cells are the end points of the endocytic pathway, they were labelled by endocytosis of horseradish peroxidase (HRP), a fluid phase marker which accumulates in lysosomes and produces an electron-dense reaction product upon exposure to its substrate, 3,3'-diaminobenzidine (DAB). Continuous bulk HRP uptake also allowed earlier components of the endocytic pathway to be labelled in these cells, including early endosomes and recycling endosomes (which could be distinguished by their characteristic tubulovesicular morphology). NK and iNKT cells were labelled overnight with HRP to load the endocytic pathway, mixed with MHC I-deficient 722.221 target cells or lipid-pulsed human CD1d-expressing C1R target cells (CD1d-C1R), respectively, and the mixed cell populations were incubated at 37C to form conjugates. Cells were fixed at different time points during target cell killing, typically between 5-60 minutes for NK and 30-70 minutes for iNKT, and prepared for EM. Thin (50 to 100 nm) sections were analysed for the position of the centrioles in NK or iNKT cells that had formed synapses with their targets.

**Figure 1:**
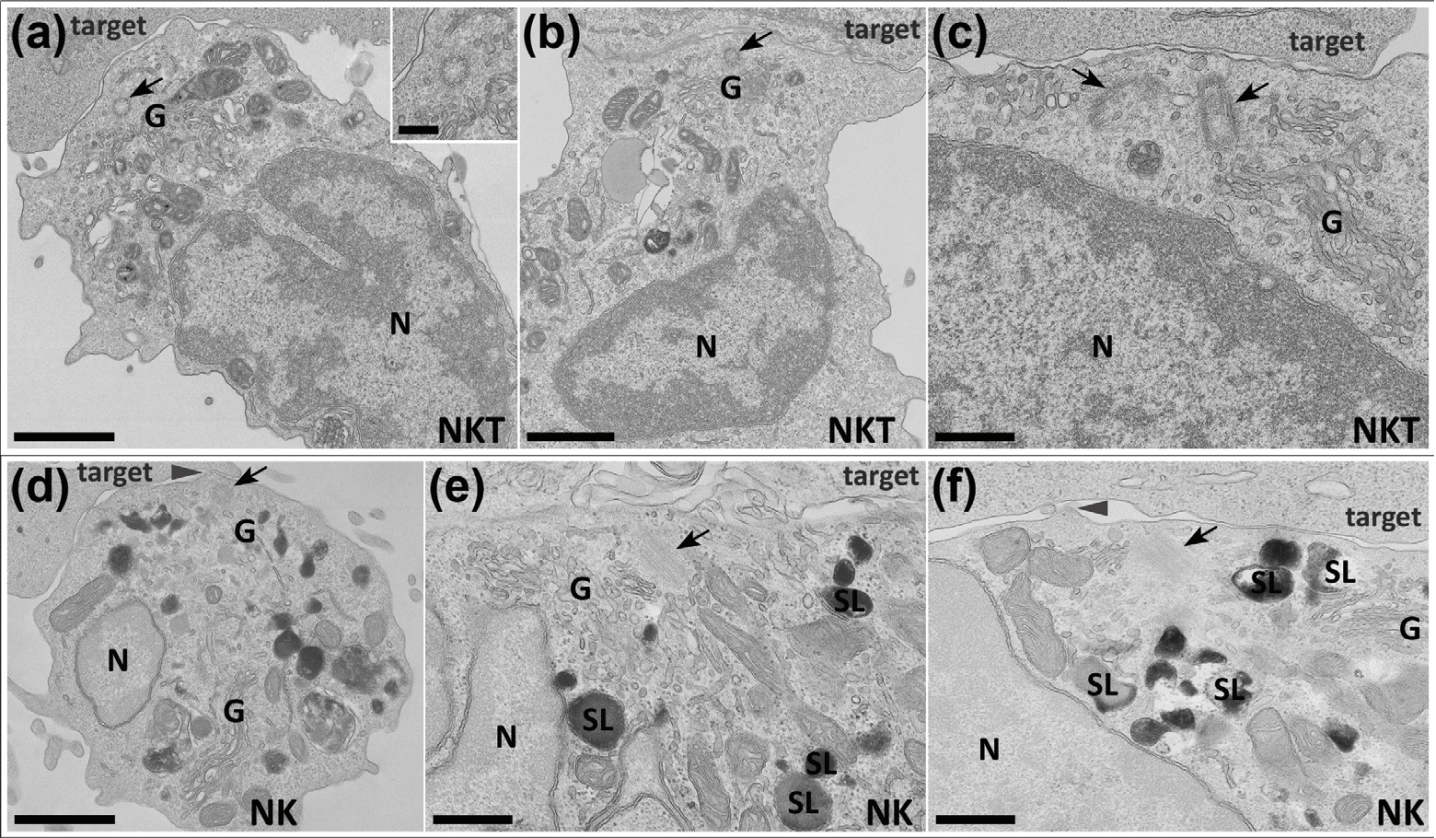
**The centrosomes of NK and iNKT cells polarise right up to the contact site membrane during target cell killing**. Low-power (**(a) **(main picture), **(b) **and **(d)**) and high-power (**(a) **(inset), **(c)**, **(e) **and **(f)**) electron microscope images of thin (50 to 100 nm) lead-stained sections of iNKT cells (**(a) **through **(c)**) and NK cells (**(d) **through **(f)**) preincubated with HRP to label the endocytic pathway processed with DAB cytochemistry to reveal electron-dense reaction products in secretory lysosomes (SL) and conjugated to either primed C1R-CD1d (**(a) **through **(c)**)or MHC I 722.221 (**(d) **through **(f)**) target cells (target) showing that the centrioles (black arrows) and accompanying microtubule organising centre (MTOC)-associated structures (for example, the Golgi complex (G)) of both iNKT and NK cells polarise right up to the plasma membrane in the centre of the lytic cell:target cell contact site to the side of the secretory cleft (SC) during target cell killing. Secretory lysosomes are distributed throughout the cell body in cells caught in the early stages of interaction with targets **(d) **but accumulate in the area surrounding the polarised centrosome as the cells prepare to kill (**(e) **and **(f)**) and are undetected in cells at late stages following release of their electron-dense content (for example, **(a) **through **(c)**). Note that polarised centrioles may be separated by some distance from the nucleus (N). Note also that small bumps and/or protrusions may appear from the cell surface opposite the polarised centrosome (for example, grey arrowheads in **(b) **and **(c)**). Bars = 1,000 nm (**(a) **(main image) and **(d)**), 500 nm (**(b)**, **(c)**, **(e) **and **(f)**) and 250 nm (**(a)**, inset).

Figure 1 shows the contact site formed between iNKT cells (Figures 1a through 1c) or NK cells (Figure 1d through 1f) and their targets as visualised by EM. The centrioles of both NK and iNKT cells have migrated to the plasma membrane at the point of contact with the target as has previously been observed for CTLs. The use of thin sections allows the greatest resolution of EM structure to be determined but does mean that only part of a single centriole will be contained within a single section. Centrioles can be seen either in cross-section (Figures 1a, b and 1d) or in longitudinal section (Figures 1c, e and 1f). The images of longitudinal sections, show clearly that centrioles can come right up to and 'dock' with the plasma membrane at the immunological synapse. As with CTLs, these tightly polarised centrioles can be some distance from the nuclear membrane (Figures 1a, b and 1d). The movement of the centriole was recognition-dependent, since no migration was seen in preparations of iNKT cells mixed with target cells that had not been lipid-pulsed. These images reveal that tight centriole polarisation to the plasma membrane is not restricted to CTLs, but also occurs in cytolytic cells of the innate immune system in response to interaction with targets.

We observed tight centriole polarisation at the immune synapse in 61 conjugates formed by NK cells and their targets, and in 88 conjugates formed by iNKT cells with targets. To investigate the frequency of centrosome polarisation at the immune synapse in more detail, we measured the distance of the centriole from the contact site in all conjugates in which the centrosome was polarised towards the target and in which at least one centriole was visible within the section plane, within a single population of cells. Since thin sections only capture part of the centriole, and since a centriole has a length of 450 nm, we considered any centriole profile within 450 nm of the cell surface to be tightly polarised, although the majority of the centrioles appeared to be much closer than this. Forty-two NK cells and fifty iNKT cells with polarised centrosomes were analysed. Both NK and iNKT cells showed similar frequencies of tight centriole polarisation at the immune synapse: 14% of NK cells and 12% of iNKT cells showed centrioles greater than 1,000 nm from the contact site, 10% of NK cells and 18% of iNKT cells had centrioles 500 to 1,000 nm from the contact site and 76% of NK cells and 70% of iNKT cells showed tight polarisation. Thus, although there was some variation in actual centriole distance, the majority of cells showed tight polarisation.

### The Golgi apparatus, secretory lysosomes and recycling endosomes polarise towards the centrosome at the plasma membrane

**Figure 2:**
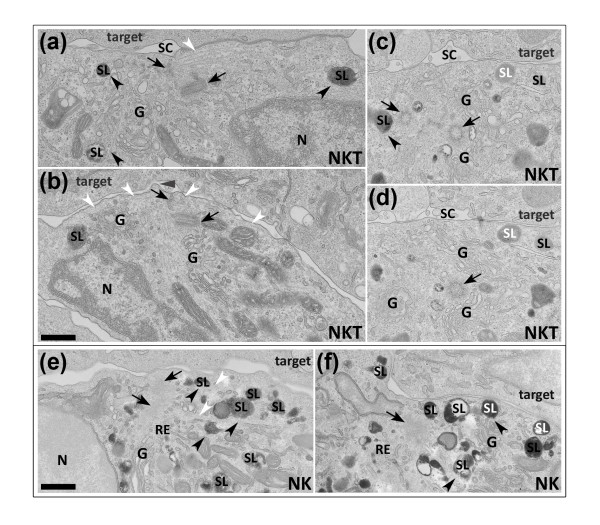
**Centrosome polarisation reorganises the microtubule network to pass under the plasma membrane at the secretory cleft and thus directs and delivers secretory lysosomes to secretory sites**. Electron microscopic images of conjugates prepared as in Figure 1 showing HRP in both secretory lysosomes (SL) and tubular endocytic recycling compartments (RE), illustrating that polarisation of the centrosome in both iNKT cells (**(a) **through **(d)**) and NK cells (**(e) **and **(f)**) during target cell (target) killing reorganises microtubules to pass under the contact site membrane (for example, white arrowheads in **(a)**, **(b) **and **(e)**), thus allowing secretory lysosomes to be directed and delivered to the secretory cleft (SC) during minus end-directed movement along microtubules towards the centrioles (black arrows). **(c) **and **(d) **show nonconsecutive serial sections through the synapse of the same cell. Black arrowheads (for example, **(a)**, **(c)**, **(e) **and **(f)**) show secretory lysosomes in contact with the microtubules. White SL labelling (for example, **(c)**, **(d) **and **(f)**) indicates secretory lysosomes docked at the contact site membrane. Grey arrow in **(b) **indicates a small cell surface protrusion projecting into the secretory cleft opposite the polarised centrosome. G = Golgi; N = nucleus. Bars = 500 nm.

Images of fixed conjugates capture the interaction of cytolytic cells with their targets at different points during their interaction, allowing different stages of killing to be analysed, although the thin (50 to 100 nm) sections required for high-resolution electron microscopic analysis limit the number of organelles that can be captured in a single plane. Figure 2 shows images of NK and iNKT cells in which both secretory lysosomes and polarised centrioles were visible in the same section plane. These images indicate that polarised secretory lysosomes accumulate near the point where the centrioles dock at the plasma membrane. In contrast, Figure 1d shows an NK cell in which the secretory lysosomes are still distributed throughout the cell and therefore is likely to represent an early stage of polarisation. Interestingly, although the secretory lysosomes have not yet polarised, the centriole is already at the contact site. This suggests that, as with CTLs [10], the centrosome can reach the contact site before the secretory lysosomes, and is consistent with the idea that centrosome polarisation and subsequent microtubule network organisation directs secretory lysosome movement to the secretory cleft.

In both NK and iNKT cells, both the Golgi complex and the endocytic recycling compartment, defined as HRP-positive structures with tubular morphologies (see, for example, Figures 2e and 2f), also polarise with the centriole to the contact site and can be seen surrounding the docked centrosome. Intriguingly, the Golgi complex moves right up to the plasma membrane in both cell types. This is particularly clear in Figure 1c for iNKT cells and in Figure 1e for NK cells, where Golgi complex-derived vesicles appear to contact the plasma membrane. These observations support the idea that constitutive secretion of proteins such as cytokines is also focused towards the immunological synapse [25-27]. Together these images suggest that, as described previously for CTLs [12,13], pathways of exocytosis and endocytosis are both focused to the point of centrosome docking at the plasma membrane in NK and iNKT cells.

### The centrosome reorganises the microtubules to pass under the secretory cleft

We have previously described a secretory cleft which appears between the CTL and its target and is the site within the immunological synapse where the secretory lysosomes dock and release their content [9,11]. Electron microscopic analysis revealed similar secretory clefts within the synapses formed between NK and iNKT cells and their targets (see, for example, Figures 2a, c, d and 2f). In Figures 2a and 2b, the centriole can be seen to contact the plasma membrane right next to the secretory cleft, with microtubules (indicated by white arrowheads) emanating from the centrosome in such a way that they pass right under the membrane along the secretory cleft. Secretory lysosomes are associated with microtubules leading towards the cleft membrane (indicated by black arrowheads in Figures 2c, d and 2f)). In this way, polarisation of the centrioles to the secretory cleft in NK and iNKT cells and the resulting reorganisation of the microtubules appear to provide a mechanism for polarised secretion by directing secretory lysosomes precisely and exclusively to the secretory cleft within the immunological synapse formed between NK and iNKT cells and their target cells as has been previously described for CTLs [4].

### The immunological synapse resembles sites of primary cilia formation

Centrosome polarisation to the plasma membrane is unusual in most cell types but is well documented during primary cilia formation, when the centrosome, or 'basal body', migrates to a site at the cell surface and forms the anchor for the elongation of microtubules which form the core of the cilia and which project out from the surface and are surrounded by a unique membrane sheath [17-20].

Our own studies on CTLs [3,4,12] have revealed striking morphological similarities between events occurring during primary cilia formation and the events occurring at the immunological synapse during CTL killing. The first and key observation was the finding that, like the primary cilia basal body, the CTL centrosome moves to a specific site on the cell membrane, where it docks [4].

We also previously noted that membrane protrusions appear opposite the site of centriole docking in the CTL synapse [12], and these resemble the protrusions observed as primary cilia form [17,18]. Intriguingly, the electron microscope images of NK and iNKT synapses presented in our present study reveal that these protrusions also occur from the plasma membrane at the synapse in these cells. Our images show that, like CTLs, these protrusions appear at the point where the centriole contacts the plasma membrane in both NK cells (for example, Figure 1d) and iNKT cells (for example, Figure 2b). Whether these protrusions are related to those formed during cilia growth or are simply part of the extensive network of interdigitations and projections that have been documented as forming between CTLs [13,28-30] and their target cells during killing has yet to be confirmed.

Another similarity between cilia and synapse formation is that both ciliary and CTL centrosomes polarise to sites on the plasma membrane opposite 'clefts': the ciliary pocket for primary cilia [21] and the secretory cleft for CTLs [9,11]. We observed that the NK and iNKT cell centrioles also polarise to the membrane opposite secretory clefts. In addition, membrane trafficking pathways (including the regulated and constitutive secretory pathways and the endocytic recycling pathway) and their associated organelles all concentrate at the site of the polarised centrosome in both cells with cilia [17,21] and in CTLs [3,12-15]. The results presented in this study mean that we can extend this comparison to NK and iNKT cells, with all these points also observed in each of these cell types in addition to CTLs.

The similarities to cilia formation are intriguing, since cells of the haematopoietic lineage, including cytolytic cells, are thought to be unable to form primary cilia [31-33] in spite of the fact that they have recently been found to express intraflagellar transport proteins involved in cilia formation [34]. We previously proposed that the similarities between the immunological synapse and cilia suggest that CTLs have exploited the mechanisms used in primary cilia formation for focusing polarised secretion from CTLs [3,4]. The observation that these features are conserved throughout several cytolytic cells, not just CTLs, supports this idea.

## Conclusions

Cytolytic cells of the immune system are very potent and efficient killers. The lethal hit delivered by releasing the contents of perforin-containing secretory lysosomes needs to be accurately focused on the target cell alone. In CTLs, a novel mechanism for directing secretion precisely via centrosomal docking at the plasma membrane has been described in which polarisation of the centrosome to contact the plasma membrane close to the secretory cleft reorganises microtubules to lie underneath the plasma membrane at this point, and directs secretory traffic to a single focused spot within the immunological synapse. In this study we have shown that NK and iNKT cells, two other cytolytic cells of the immune system, use the same mechanism as CTLs for rapid and precise secretion. These findings extend our initial proposal that the immunological synapse resembles the site of cilia formation and point to a common mechanism for polarised secretion from cytolytic cells of the immune system.

## Methods

### Culture and preparation of lytic and target cells for conjugation

Human NK cells from volunteer healthy donors were purified using the RosetteSep method (StemCell Technologies, Vancouver, BC, Canada) and cultured on irradiated feeder cells in the presence of 2 μg/mL phytohemagglutinin (Life Technologies, Paisley, UK) and 100 U/mL recombinant human IL-2 (Proleukin; Chiron Corp., Emeryville, CA, USA) to obtain proliferation of polyclonal NK cells. All blood samples from healthy donors were obtained with their informed consent. Epstein-Barr virus-transformed human B cells (721.221 cell line lacking MHC I) were grown and maintained in culture in RPMI 1640 medium containing 10% FCS, and were used as targets for NK cells.

Human iNKT cells were expanded from healthy donors' peripheral blood lymphocytes (PBL) with autologous dendritic cells pulsed with α-galactosylceramide (α-GalCer) (provided by Prof GS Besra, School of Biosciences, University of Birmingham, Edgbaston, Birmingham, UK) as described previously [24]. Two weeks after priming, iNKT cell lines were isolated by cell sorting with CD1d-α-GalCer tetramers and anti-Vα24 and anti-Vβ11 antibodies (Immunotech, Marseille, France). iNKT cells were grown in complete medium supplemented with 5% human serum instead of 10% FCS, and 1,000 U/ml IL-2, and were periodically restimulated with allogeneic PBL as described previously [35]. C1R-CD1d cells were used as targets for iNKT cells and were grown in complete medium (RPMI 1640 medium containing 10% FCS, penicillin/streptomycin, glutamine, nonessential amino acids and sodium pyruvate).

### Preparation of lytic and target cells for conjugation

To allow compartments of the endocytic pathway (including secretory lysosomes and recycling endosomes) to be identified in lytic cells during killing events, NK and iNKT cells were incubated for 14 to 16 hours immediately before conjugation, in 1 mg/mL HRP added directly to the growth medium as described previously [9,36]. C1R-CD1d target cells were either primed for iNKT cell recognition by pulsing the target cells overnight with 1 mg/mL α-GalCer in 150 mmol NaCl and 0.5% Tween 20 (pulsed) or incubated in 150 mmol NaCl and 0.5% Tween 20 alone (unpulsed, control C1R-CD1d cells). All cells were washed at least three times in serum-free growth medium to remove serum and excess HRP (lytic cells) or priming ligand (target cells), resuspended at a concentration of 1 × 10^6 ^cells/mL in growth medium lacking serum and used immediately for conjugation.

### Conjugation of lytic cells to target cells and fixation and preparation of samples for EM

Lytic (NK or iNKT) cells were mixed 1:1 with appropriate targets, plated immediately into 24-well plates (0.5 mL/well) and incubated at 37°C for 5, 10, 20, 40 or 60 minutes (NK cells) or for 30 to 40 minutes or 60 to 70 minutes (iNKT cells) to allow cells to form lytic cell-target cell conjugates. Four separate conjugation experiments were performed for each cell type. iNKT cell samples were prepared in duplicate and three separate donors were used to provide NK cells. Similar results were observed for each experiment. Conjugates were fixed by the addition of 2× fixative (3% gluteraldehyde and 4% paraformaldehyde in PBS) directly into the incubation medium and left for 10 minutes at room temperature. Fresh 1× fixative (1.5% gluteraldehyde and 2% paraformaldehyde in PBS) was added and the samples were incubated for an additional 40 to 50 minutes at room temperature before being processed for DAB cytochemistry as described previously [36]. NK cell conjugates were postfixed with 1% osmium tetroxide containing 1.5% potassium ferricyanide for 1 hour at room temperature [36]. iNKT cell conjugates were postfixed with 1% osmium tetroxide alone followed by *en bloc *staining in uranyl acetate as described previously [10]. Sections (50 to 100 nm) were stained with lead citrate and viewed using Philips Tecnai G2 or C100 transmission electron microscopes (FEI UK Limited, Cambridge, UK). Images were captured using photographic negative film (Kodak Ltd., Hemel Hempstead, UK) and scanned using a Hasselblad Flextight X5 scanner (Hasselblad UK Ltd, Centennial Park Herts, UK).

## Abbreviations

FCS: foetal calf serum; IL: interleukin; PBS: phosphate-buffered saline.

## Competing interests

The authors declare that they have no competing interests.

## Authors' contributions

DP and MS generated and provided NK and iNKT cells, respectively. MS prepared iNKT and C1R-CD1d cells for conjugation. JCS performed all cell conjugation experiments, preparation of samples for electron microscopy and analysis by electron microscopy. JCS, GG and MS designed and discussed the experiments and results. JCS and GG wrote the manuscript. All authors read and approved the final manuscript.
